# The Perception of COVID-19 among Italian Dentists: An Orthodontic Point of View

**DOI:** 10.3390/ijerph17124384

**Published:** 2020-06-18

**Authors:** Stefano Martina, Alessandra Amato, Roberto Rongo, Mario Caggiano, Massimo Amato

**Affiliations:** 1 Department of Medicine, Surgery and Dentistry Scuola Medica Salernitana, University of Salerno, 84081 Baronissi, Italy; smartina@unisa.it (S.M.); aamato@unisa.it (A.A.); macaggiano@unisa.it (M.C.); mamato@unisa.it (M.A.); 2 Discipline of Orthodontics, Department of Neurosciences, Reproductive Sciences and Oral Sciences, University of Naples Federico II, 80131 Napoli, Italy

**Keywords:** COVID-19, dentistry, orthodontics, anxiety, risk management, survey

## Abstract

COVID-19 has severely impacted dentists, who are at a great risk of infection. This study aimed to investigate if dentists are anxious about returning to their daily activities, and what the perception of the risk is for dentists and orthodontists regarding orthodontic procedures. An online questionnaire, including the Patient Health Questionnaire-4 (PHQ-4), was sent to Italian dentists during the final days of the lockdown with items about anxiety, fear, distress, perceived risk for operators, and concerns about orthodontic patients caused by working during the COVID-19 outbreak. Data were analyzed with a chi-square test and logistic regression analysis. The level of significance was set as *p <* 0.05. A total of 349 dentists completed the survey, including 183 orthodontists. Returning to their daily work activity was a source of anxiety for 192 participants and this was associated with the level of distress (odds ratio (OR) = 3.7; *p <* 0.001). Most of the orthodontists (67.6%) thought that they would increase the number of working hours during the week (OR = 1.8; *p =* 0.007). Italian dentists were mostly scared to return to their daily activities because they considered their jobs a high risk to them and their families. Dentists with an exclusive/prevailing orthodontic activity were forced to increase their working day during the week.

## 1. Introduction

In December 2019 a novel coronavirus (2019-nCoV) was described in the Chinese city of Wuhan [1]. The 2019-nCoV, afterwards renamed SARS-CoV-2 [2], caused a highly contagious disease (COVID-19) [3]. COVID-19 patients could have upper respiratory tract infection (RTI), fever, dry cough, dyspnea and severe viral pneumonia with respiratory failure and even death, but there is a percentage of those infected that shows mild symptoms (pharyngodynia, nasal congestion, olfactory and gustative disorders) or no symptoms [4,5]. COVID-19 could directly cause cardiovascular injuries such as pericarditis, myocarditis, myocardial infarction, heart failure, arrhythmias or thromboembolic events [6]. In a few weeks, COVID-19 had spread all over the world and on 11 March 2020, the World Health Organization (WHO) declared it a pandemic. The virus propagates between people through close contact and via respiratory droplets produced from coughs or sneezes [7], but several uncertainties currently remain, particularly regarding the contribution of asymptomatic versus symptomatic patients to the transmission of the virus [8]. Virus identification in human respiratory epithelial cells could be positive about 96 h from exposure and 24–48 h before the onset of symptoms [9]. The nasopharyngeal real-time polymerase chain reaction (RT-PCR) swab test was used to diagnose COVID-19. However, this test has high costs and complex execution methods and, even though one of the epidemic fight strategies is the identification and isolation of the positive subject, the nasopharyngeal swabs on the whole population is still not sustainable. The available diagnostic accuracy data suggest that the test has very high specificity, but a moderate sensitivity (63–78%) [10]. In the absence of a vaccine or an effective treatment, many countries including Italy have adopted quarantine measures and lockdowns to control the spread of COVID-19, limiting mortality and maintaining healthcare demands within capacity. On 1 May in Italy 207,428 cases of COVID-19 were diagnosed out of 1,398,633 subjects tested, with 28,236 deaths [11].

In Italy, during the lockdown (also known as phase-1), the dental assistance in private practices was not suspended by law to reduce the burden on the hospitals dealing with the COVID-19 patients. However, the national medical and dental committee and the category unions strongly recommended rescheduling dental activities, limiting these to emergencies, such as pulpitis, abscesses and phlegmons. It can be assumed that stopping dental procedures may be due to several reasons: to avoid the movement of people, to reduce the consumption of personal protective equipment (PPE) and to reduce the risk of infection. Indeed, aerosols formed during dental procedures, contact spread, and contaminated surface spread are possible routes of transmission for SARS-CoV-2 [12]. For these considerations, most of the treatments, including routine orthodontic appointments, were postponed. Orthodontic patients were assisted virtually by means of photos, videos, or video calls, performing triage to select the real urgencies to be managed in person from remotely manageable situations [13].

The lockdown ended in Italy on 4 May and the so-called phase-2 has started by imposing social distancing measures and the use of masks in everyday life. In a dental practice, it is obviously impossible to maintain these measures; therefore, dentists have a high risk of getting infected from patients and potentially spreading the virus. In light of this, it is not surprising for dentists to be afraid of being infected by their patients. On the other hand, orthodontics could be a less risky practice than other specialties of dentistry. Orthodontic controls are usually quick, aerosol procedures are few and not very frequent during the same clinical day, and most patients are under 20 years of age, a category that seems to be less affected by COVID-19 [14].

Previous studies investigated the level of fear and psychological distress during the COVID-19 pandemic [15,16], finding that elevated psychological distress was present among dentists who have a background illness, those who feared contracting COVID-19 from a patient, and those who had higher subjective overload. None of these studies were performed in Italy.

The purpose of this study was to investigate how the return to their daily practice during phase-2 influences dentists and what their perception of risk is regarding orthodontic procedures.

## 2. Materials and Methods

The present cross-sectional study was conducted by sending an online survey questionnaire to Italian dentists, from 1 May to 6 May 2020. The survey was created on an online survey development cloud-based software called SurveyMonkey^®^ (SVMK, San Mateo, CA, USA). The participants were approached via Facebook, WhatsApp, and mailing lists. Participants authorized the treatment of their data that were collected anonymously. The personal information of participants was protected and there were no inhumane questions or investigations. The questionnaire was comprised of 31 multiple-choice questions: 5 questions were about personal data (gender, age, region of residence); 4 were on symptoms of COVID-19; 7 were about the perceived risk for operators during orthodontic procedures, anxiety and distress caused by working during the COVID-19 outbreak; 6 were about the fears for an infection and the concerns regarding orthodontic and Temporomandibular Disorders (TMD) patients whose treatment has been suspended due to the epidemic; 5 were about emergencies and changes in clinical procedures and work organization as a result of the COVID-19. The last 4 questions of the Patient Health Questionnaire (PHQ-4) [17] were included in the questionnaire to assess whether the general state of anxiety and depression could influence the other answers. The PHQ-4 was composed of four questions with a 4-item Likert scale. Scores between 3 and 5 indicated a slight distress, between 6 and 8 indicated a moderate psychological distress, and higher than 10, a severe distress. In the survey, most of the questions had a dichotomous answer (yes/no). Dentists were asked about the number of emergencies they faced during the lockdown period (less than 5, more than 5) and they had to classify what they feared most in the case of infection among the following: fear of dying, fear of the quarantine, fear of infecting family members, fear of economic loss, fear of interrupting treatments, or fear of discrimination.

### Statistical Analysis

Frequencies and percentages for categorical data were computed. A chi-square test was used to assess the association between gender (male vs. female), age (older than 70 years old vs. 60–69 vs. 50–59 vs. 40–49 vs. 30–39 vs. 20–29), region of residence (South vs. Centre-North), level of distress (0–1 vs. 2–3), orthodontic activity (general practice vs. main/exclusively orthodontic activity) and questions about risk, anxiety and distress caused by working during the COVID-19 outbreak. Furthermore, the chi-square test was used to evaluate the association between the level of distress and the orthodontic activity with the 5 questions about emergencies and changes in clinical procedures and work organization as a result of the COVID-19. In the case of a statistically significant association, logistic regression analyses to calculate the odds ratio (OR) were performed. A standard statistical software package (SPSS, version 22.0; SPSS IBM, Armonk, New York, USA) was used. The level of significance was set at *p* < 0.05.

## 3. Results

A total of 349 (175 M, 173 F, 1 Not Reported) dentists completed the survey. Participants were asked to identify their age range and the most common were 30–39 years old (110) and 50–59 years old (76). Most of the dentists lived with their families (289) or alone (51). A total of 226 were from the South of Italy and 123 from the Centre-North Italy. Among the 349 participants, 183 had a main (91) or exclusive (92) orthodontic practice, while 166 had a more general practice. None of the participants were diagnosed as COVID-positive but only 35 were tested. The PHQ-4 reported 4 different levels of distress: 112 participants reported no distress, 160 slight distress, 58 moderate distress, and 19 severe distress. These data are shown in the Table 1.

The level of distress was associated with 2 questions about anxiety and distress; indeed, 78% (60/77) of the dentists with higher distress levels were more anxious about starting their daily work activities again (odds ratio (OR) = 3.7; *p* < 0.001) compared with 48.7% (132/271) of the dentists with less distress. Furthermore, 31.2% (24/77) of dentists with higher distress levels were more inclined to close their activities (OR = 3.9; *p* < 0.001) compared with 10.3% (28/271) of participants with less distress, for a total of 52 subjects out of 349 that were more inclined to stop their jobs (Table 2 and Table 3).

Only 27 out of 349 participants did not consider their work to be at a higher risk, and only 42 did not consider their work to be of higher risk also for their families. Among these 42, 28% (12/42) were younger than 40 years old (OR = 6.1; *p* = 0.002) and 66% (28/42) were from Centre-North Italy (OR = 0.4; *p* = 0.004). Returning to daily work activities was a source of anxiety for 192 participants, mostly women; indeed, 64% of women (110) replied yes against only 46.9% of men (82) (OR = 2; *p* = 0.003). Similarly, this was a source of anxiety more for those participants who were younger than 50 years old (141) than for those older (51), (OR = 2.6; *p* = 0.026). Regarding teamwork, 56 out of 349 dentists thought that their collaborators had less risk than dentists of being infected by COVID-19. Most of the participants (221) considered orthodontic procedures as a lower risk of contagion than general dental practices, even if adolescents and children were mostly considered a similar source of infection as adults (Table 2 and Table 3).

The survey also asked about the number of emergencies faced during the lockdown period and 148 dentists had more than 5 patients during this lockdown period, but 79 dentists were extremely scared and 157 were moderately scared of dealing with emergencies. Interestingly, there was an association between the number of dentists that dealt with fewer than 5 emergencies during this period and the level of fear facing the emergencies with 62.2% (147/236) of dentists that were strongly or moderately scared who faced less than 5 emergencies compared with 52.3% (59/112) of dentists that were slightly or not scared that faced more than 5 emergencies (OR = 1.8; *p* = 0.035). Finally, there was an association between the level of distress (PHQ-4) and the fear during emergencies. Indeed, among the 77 dentists that had a high level of distress, 48% (37) were strongly scared and only 4% (3) were unafraid (OR = 0.1; *p* < 0.001) (Table 4 and Table 5).

Furthermore, dentists thought that patients under fixed treatment who were not controlled for more than two months should be considered emergencies (209), while only 104 and 117 dentists considered emergencies those patients with removable appliances or with temporomandibular disorders that had not been controlled for 2 months (Table 4).

Finally, most of the dentists (342) thought that in this phase-2 they would reduce the number of patients during the day and 211 thought that they would increase their number of working hours during the week. Interestingly, among these 211, the greater percentage (58.3%, 123/211) were dentists who had a main/exclusive orthodontic activity (OR = 1.8; *p* = 0.007), and similarly among this group of dentists there was the greater percentage of participants that planned to postpone aerosol procedures (60.7%, 105/173, OR = 1.9; *p* = 0.002).

Regarding the ranking of fears in case of contagion, most of the participants were afraid of infecting family members (indicated 160 times as their first fear), dying (indicated 82 times as their first fear), and being discriminated against (indicated 31 times as their first fear, Table 6).

## 4. Discussion

This study was carried out during the final days of the lockdown in Italy and just before dentists returned to their practices. The aim was to understand through a survey how dentists had approached phase-1 and how they were preparing for phase-2 and investigate whether these situations had created anxiety or distress.

The sample was a good representation of different age ranges, different regions of residence, different levels of distress and different dental activities. None of the interviewed dentists were positive for COVID-19 and almost 10% were tested. This percentage is higher than the total number of swabs done in Italy (by 9 May, 4% of the population). In Italy there was a considerable difference in the incidence of COVID-19 between central-northern and southern Italy, with nearly 90% of total cases from the Centre-North [11]. Therefore, the answers of dentists from the Centre-North were compared with those from the South. Despite the different number of cases, the perception of the problem by dentists between Centre-North and South was similar. This could be due to the quarantine measures which were the same in all regions of Italy and to the influence of the Italian media which stressed the situation without any differences during phase-1.

The PHQ-4 showed that 77 dentists out of 349 (22.1%) had moderate/severe psychological distress (score equal to or higher than 6) in the last 2 weeks. This percentage was greater compared with the data presented of the general population, where about 5% of the population presented these scores [18]. It could be argued that the tough situation due to the pandemic crisis and the possible Italian economic crisis might influence the psychological status of the participants. Moreover, it was interesting that people with higher distress levels were those with more anxiety about returning to work, more inclined to close their activities, more afraid of facing emergencies, and more fearful of infecting their families. This critical situation has highlighted the difficulties that Italian dentists had to face during their activities, and as a vicious circle in which more psychological distress led to more fear and anxiety, that led to a decrease in psychological wellness [19].

Most dentists interviewed considered themselves and their collaborators to be exposed to a high risk due to their jobs and they are afraid of infecting their relatives. These data are consistent with previous studies on the psychological impact in health care workers of similar infectious diseases, such as severe acute respiratory syndrome (SARS), who were afraid of contracting an infection while treating an infected patient or infecting a family member [20,21]. However, some associations were found between dentists that did not think they were a higher source of infection risk for their relatives due to their jobs regarding age and region of residence. This is probably due to the fact that, although with different means of contagion, dentists with more experience are used to managing in their practice several infective diseases, such as hepatitis B, hepatitis C, human immunodeficiency virus infection, tuberculosis, following strict protocols for infection control [22].

Although many dentists treated less than 5 patients in phase-1, the management of these emergencies frightened them. The fear in dealing with emergencies was associated with the distress level of the dentists (the higher the distress, the higher the level of fear) but also with the number of emergencies seen during the lockdown period. Indeed, the dentists that were more scared in facing the emergencies were those that treated fewer than 5 emergencies. Two hypotheses might explain this association: first, dentists who were more fearful tried as much as possible to solve any potential emergency by using telemedicine to avoid meeting patients. Second, those dentists who performed more than 5 emergencies understood that with adequate PPE the chance of contagion was low. However, due to the lack of PPE during phase 1 in Italy, the fear of performing any emergency procedure may have also been due to the lack of availability of proper PPE. 

Fifty-five percent of respondents were worried about the return to their dental practices in phase-2; 15% contemplated definitively ceasing their activity. It is obvious considering that, since there is no vaccine or approved treatment, anxiety about being infected is very high in the population. Dentists and health professionals in general are at higher risk of contracting infectious diseases, with an additional psychological distress component [23]. Moreover, dentists may be concerned about liability related to possible infection of workers or patients in their practices, and about the critical Italian economic situation which could reduce the demand for dental treatment. Women, dentists younger than 50 years old, and participants with higher distress levels were more concerned about coming back to work compared with other categories. As already mentioned, psychological distress and the fear of coming back to work with an increased patient load strongly influenced each other due to the concern about the contagion, and probably women and young people were more worried about their families. Indeed, when asked to rank the situations that would be most frightening in case of infection, most dentists ranked the fear of infecting their relatives and the fear of dying as the top two. Few have attributed importance to the possibilities of being discriminated against, economic loss and the suspension of ongoing treatments. Surprisingly, dentists from the South said they were more concerned with infecting family members.

In addition, most dentists believed that children and adolescents represent the same risk of infection compared with adults.

A part of the questionnaire was focused on orthodontics and about 50% of the participants were orthodontists. Most dentists thought that, with orthodontics, the risk of being infected is lower. The explanation for this could be that, unlike other branches of dentistry, in orthodontics there are procedures that do not require aerosols. Aerosol procedures were indicated as more infective procedures in several articles [24,25]. In fact, most orthodontists (57%) declared that they will postpone clinical procedures with aerosols as opposed to general practitioners, in which only 40% will postpone these procedures. Sixty percent of participants believed that not examining a patient on fixed appliance treatment for more than 2 months is a serious problem, whereas they believed it is less critical to postpone exams for patients who have removable devices. Some orthodontists may be worried about orthodontic activations of fixed appliances given before the lockdown, which hardly can be monitored with telemedicine. Other orthodontists may believe that not checking patients for more than 2 months may have caused damage to the appliance, such as loose brackets and bands or worsened oral hygiene. These are all patient cooperation factors that could contribute significantly to increased treatment time [26]. Removable appliances may suffer less from these issues and generally cause less discomfort to patients as compared with fixed appliances [27]. Regarding rescheduling patients, compared with general practitioners a higher percentage of orthodontists declared that they would organize their weekly schedules to work more days during the week (53.0% vs. 67.2%). This is an important change in orthodontic practice because in Italy most dentists that have a main/exclusive orthodontic activity work as consultants. Due to the greater time needed to perform disinfection procedures, the chair time between two patients will increase and the number of patients that is possible to see in one day, obviously, will decrease forcing the dentists to work more days per week.

Regarding the management of TMD patients, most dentists thought that examining these patients is not an emergency; however, they believed that not controlling them for more than 2 months may have worsened the patient’s psychological distress.

This study presents some limitations. It is a survey-based study; hence information is self-reported, and the collection time was only one week. However, it also presents some strengths, such as the high number of participants, the good distribution of the factors, and the focus on the orthodontic practice.

## 5. Conclusions

This survey-based study investigated the perception of Italian dentists of the return to their daily practices and of their risk during orthodontic procedures.

Italian dentists were mostly afraid to return to their daily activities, and women, younger dentists and dentists with higher distress levels were more frequently scared by this aspect.

The worst fears in case of contagion was infecting family members and the fear of dying.

Dentists considered their job a high risk to them and their families; however, orthodontic procedures were considered a lower risk compared with general dental procedures. 

Dentists with an exclusive/main orthodontic activity were forced to increase their working day during the week.

## Figures and Tables

**Table 1:** General data of the participants.

Variable	Frequency (Percentage)					
Age	20–29 65 (18.6%)	30–39 110 (31.5%)	40–49 57 (16.3%)	50–59 76 (21.8%)	60–69 35 (10%)	>70 6 (1.7%)
Gender	Males 175 (50.1%)			Females 173 (49.9%)		
Region of residence	Centre-North 123 (35.2%)			South 226 (64.8%)		
Orthodontic activity	Exclusive/main 183 (53.5%)			General practice 166 (47.5%)		
Tested for COVID-19	Yes 35 (10%)			No 314 (90%)		
PHQ-4	No/low distress 272 (78%)			Moderate/high distress 77 (22%)		

**Table 2:** Frequencies and percentages of replies to perceived risk, anxiety, and distress questions.

Survey Question	Frequency (Percentage)			
Consider dental practice as at higher risk	Yes 322 (92.3%)		No 27 (7.7%)	
Consider lower risk for dental assistants	Yes 56 (16.1%)		No 293 (83.9%)	
Consider orthodontic procedures at lower risk	Yes 221 (63.1%)		No 128 (36.9%)	
Consider their work of higher risk for their families	Yes 307 (88%)		No 42 (12%)	
Anxiety to come back to working daily activity	Yes 192 (55.2%)		No 156 (44.8%)	
Consider ceasing their activity definitively	Yes 52 (14.9%)		No 297 (85.1%)	
Consider children or adolescents as a risk	Higher 58 (16.7%)	Equal 222 (63.8%)		Lower 68 (19.5%)

**Table 3:** Chi-square test and regression analysis for perceived risk, anxiety, and distress questions and gender, age, residence region, orthodontic activity, PHQ-4.

Survey Question	Gender		Age		Residence Region	Orthodontic Activity		PHQ-4	
	Χ^2^	OR (95% CI)	Χ^2^	OR (95% CI)	Χ^2^	OR (95% CI)	Χ^2^	Χ^2^	OR (95% CI)
Consider dental practice as at higher risk	0.933		0.094		0.154		0.950	0.235	
Consider lower risk for dental assistants	0.566		0.062		0.166		0.385	0.105	
Consider orthodontic procedures at lower risk	0.389		0.361		0.287		0.807	0.652	
Consider children or adolescents as a risk	0.115		0.066		0.087		0.074	0.162	
Consider their job of higher risk for their families	0.595		0.002	6.1 (1.5;24.9)	0.004	0.4 (0.2;0.7)	0.327	0.916	
Anxiety to come back to working daily activity	0.003	2 (1.3;3)	0.034	2.6 (1.1;6.1)	0.073		0.929	<0.001	3.7 (2.1;6.7)
Consider ceasing their activity definitively	0.855		0.733		0.565		0.207	<0.001	3.9 (2.1;7.3)

Χ^2^, *p*-value of the chi-squared test; OR, odds ratio of the logistic regression model; 95% CI, 95% confidence interval of the odds ratio.

**Table 4:** Frequencies and percentages of replies to questions about orthodontic and TMD patients, emergencies and changes in clinical procedures and work organization.

Survey Question	Frequency (Percentage)	
Consider fixed orthodontic treatments as emergencies after lockdown	Yes 209 (59.9%)	No 140 (40.1%)
Consider removable orthodontic treatments as emergencies after lockdown	Yes 104 (29.8%)	No 245 (70.2%)
Consider TMD patients as emergencies after lockdown	Yes 117 (33.5%)	No 232 (66.5%)
Think that TMD patients could have worsened without controls	Yes 211 (60.4%)	No 138 (39.6%)
Been contacted by TMD patients during lockdown	Yes 81 (23.1%)	No 268 (76.9%)
Postpone orthodontic procedures with aerosols	Yes 173 (49.6%)	No 176 (50.4%)
Reduce the number of patients per day	Yes 342 (98%)	No 7 (2%)
Increase the number of working hours per week	Yes 210 (60.2%)	No 139 (39.8%)
Number of emergencies during lockdown	<5 201 (57.6%)	>5 148 (42.4%)
Scared during emergencies management	Yes 236 (67.6%)	A little/No 113 (32.4%)

**Table 5:** Chi-square test and regression analysis for questions about emergencies and changes in clinical procedures and work organization and orthodontic activity, PHQ-4.

Survey Question	Number of Emergencies during Lockdown		Orthodontic Activity		PHQ-4	
	Χ^2^	OR (95% CI)	Χ^2^	OR (95% CI)	Χ^2^	OR (95% CI)
Postpone orthodontic procedures with aerosols			0.002	1.9 (1.3;2.9)	0.619	
Reduce the number of patients per day			0.801		0.877	
Increase the number of working hours per week			0.007	1.8 (1.2;2.8)	0.724	
Number of emergencies during lockdown			0.060		0.018	8.1 (1.1;64.6)
Scared during emergencies management	**0.035**	1.8 (1.2;2.9)	0.290		<0.001	0.1 (0.04;0.5)

Χ^2^ *p*-value of the chi-squared test; OR, odds ratio of the logistic regression model; 95% CI, 95% confidence interval of the odds ratio.

**Table 6:** Ranking of participants for fears in case of infection.

Fear	1	2	3	4	5	6	SCORE
Fear to infect familiars	160 (48.2%)	67 (20.2%)	17 (5.1%)	13 (3.9%)	21 (6.3%)	54 (16.3%)	4.51
Fear of die	82 (26.6%)	83 (26.9%)	35 (11.4%)	27 (8.8%)	28 (9.1%)	53 (17.2%)	4.02
Fear of being quarantined	22 (7.3%)	50 (16.6%)	64 (21.2%)	64 (21.2%)	69 (22.8%)	33 (10.9%)	3.31
Fear of economic loss	14 (4.4%)	42 (13.1%)	93 (29%)	78 (24.3%)	65 (20.2%)	29 (9%)	3.30
Fear of suspending ongoing treatments	21 (6.4%)	42 (12.9%)	61 (18.7%)	70 (21.5%)	76 (23.3%)	56 (17.2%)	3.06
Fear to be discriminated	31 (9.3%)	31 (9.3%)	47 (14.1%)	62 (18.6%)	57 (17.1%)	105 (31.6%)	2.80
